# Supplementary material for: Correlates of bullying victimization among school adolescents in Nepal: Findings from 2015 Global School-Based Student Health Survey Nepal
Source: PLoS One. 2020 Aug 19;15(8):e0237406. doi: 10.1371/journal.pone.0237406 (PMC7444580; doi:10.1371/journal.pone.0237406)
Supplement: S1 Table — (PDF) [file pone.0237406.s001.pdf]

Table S1. Description of Independent variables used in the study.

Variable	Questions	Code
Age	How old are you?	1=11 to 14 years (Early adolescent)<br>2=15 years and above (Late adolescent)
Sex	What is your sex?	1=Male<br>2=Female
Felt lonely	During the past 12 months, how often have you felt lonely?	1=Sometimes, most of the time, always<br>0=Never, rarely
Anxiety	During the past 12 months, how often have you been so worried about something that you could not sleep at night?	1=Sometimes, most of the time, always<br>0=Never, rarely
Considered suicide	During the past 12 months, did you ever seriously consider attempting suicide?	1=Yes<br>2=No
Attempted suicide	During the past 12 months, how many times did you actually attempt suicide?	1= 1 or more times<br>0= 0 times
Involved in physical fight	During the past 12 months, how many times were you in a physical fight?	1= 1 or more times<br>0= 0 times
Didn't go to school due to unsafe	During the past 30 days, on how many days did you not go to school because you felt you would be unsafe at school or on your way to or from school?	1= 1 or more days<br>0= 0 days
Missed school without permission (Truancy)	During the past 30 days, on how many days did you miss classes or school without permission?	1= 1 or more days<br>0= 0 days

Smoking	During the past 30 days, on how many days did you smoke cigarettes?	1= 1 or more days<br>0= 0 days
Smokeless tobacco use	During the past 30 days, on how many days did you use any tobacco products other than cigarettes, such as chewing tobacco surti, khaini, gutka, or parag?	1= 1 or more days<br>0= 0 days
Alcohol use	During the past 30 days, on how many days did you have at least one drink containing alcohol?	1= 1 or more days<br>0= 0 days
Overweight	How tall are you without your shoes on?<br>How much do you weigh without your shoes on?	
Underweight	How tall are you without your shoes on?<br>How much do you weigh without your shoes on?	
Sexual risk behavior	During your life, with how many people have you had sexual intercourse?	1= 2 or more people<br>0= never had sexual intercourse, 1 person
Physically active	During the past 7 days, on how many days were you physically active for a total of at least 60 minutes per day?	1= 7 days<br>0= 0 to 6 days
Bullied	During the past 30 days, on how many days were you bullied?	1= 1 or more times<br>0= 0 times
