# Supplementary material for: Correlates of bullying victimization among school adolescents in Nepal: Findings from 2015 Global School-Based Student Health Survey Nepal
Source: PLoS One. 2020 Aug 19;15(8):e0237406. doi: 10.1371/journal.pone.0237406 (PMC7444580; doi:10.1371/journal.pone.0237406)
Supplement: S2 Table — (PDF) [file pone.0237406.s002.pdf]

Table S2. Characteristics of research participants.

	Male		Female		Total	
	**N**	**%(95%CI)**	**N**	**%(95%CI)**	**N**	**%(95%CI)**
**Age**						
Early adolescent	1452	53.3(50.2,56.4)	1784	58.7(54,63.3)	3236	56.1(52.7,59.4)
Late adolescent	1551	46.7(43.6,49.8)	1613	41.3(36.8,46)	3164	43.9(40.6,47.3)
**Felt lonely**						
Never	1957	66.9(62.7,70.8)	2144	65.9(63.3,68.4)	4101	66.4(63.7,68.9)
Ever	1009	33.1(29.2,37.3)	1177	34.1(31.7,36.7)	2186	33.6(31.1,36.3)
**Anxiety**						
Never	2039	68.2(64.4,71.8)	2044	63.5(59.8,67.1)	4083	65.8(62.6,68.9)
Ever	963	31.8(28.2,35.6)	1335	36.5(32.9,40.2)	2298	34.2(31.1,37.4)
**Considered suicide**						
No	2611	86.7(83.3,89.6)	2897	86.1(83,88.7)	5508	86.4(83.8,88.6)
Yes	343	13.3(10.4,16.7)	438	13.9(11.3,17)	781	13.6(11.4,16.2)
**Attempted suicide**						
No	2758	90.3(87.2,92.7)	3061	89.1(86.2,91.5)	5819	89.7(86.9,91.9)
Yes	248	9.7(7.3,12.8)	324	10.9(8.5,13.8)	572	10.3(8.1,13.1)
**Involved in**						

**physical fight**						
No	1750	56.3(52.6,60)	2239	65.2(60.9,69.2)	3989	60.9(57.7,63.9)
Yes	1260	43.7(40,47.4)	1153	34.9(30.8,39.1)	2413	39.2(36.1,42.3)
**Didn't go to school due to unsafe**						
No	1772	58(53.1,62.8)	2122	59.6(52.7,66.2)	3894	58.8(53.2,64.3)
Yes	1215	42(37.2,46.9)	1234	40.4(33.8,47.3)	2449	41.2(35.7,46.8)
**Missed school without permission (Truancy)**						
No	2142	71.9(67.6,75.9)	2392	71.5(66.6,75.9)	4534	71.7(67.7,75.3)
Yes	814	28.1(24.1,32.4)	908	28.5(24.1,33.4)	1722	28.3(24.7,32.3)
**Smoking**						
No	2703	91.1(88.5,93.1)	3236	96.5(94.6,97.7)	5939	93.8(92,95.3)
Yes	251	8.9(6.9,11.5)	91	3.5(2.3,5.4)	342	6.2(4.7,8)
**Smokeless tobacco use**						
No	2803	92.4(89.9,94.4)	3273	95.8(93.7,97.2)	6076	94.2(92.1,95.7)
Yes	205	7.6(5.6,10.1)	110	4.2(2.8,6.3)	315	5.8(4.3,7.9)
**Alcohol use**						
No	2765	92.9(91.1,94.4)	3231	96.3(94.7,97.5)	5996	94.7(93.1,95.9)

Yes	191	7.1(5.6,8.9)	98	3.7(2.5,5.3)	289	5.3(4.1,6.9)
**Overweight**						
No	2847	93.4(90.1,95.6)	3239	94.8(92.8,96.3)	6086	94.1(91.7,95.9)
Yes	169	6.6(4.4,9.9)	167	5.2(3.7,7.2)	336	5.9(4.1,8.3)
**Underweight**						
No	2309	86.2(82.5,89.2)	2854	91.9(89.7,93.7)	5163	89.1(86.4,91.4)
Yes	384	13.8(10.8,17.5)	244	8.1(6.3,10.3)	628	10.9(8.6,13.6)
**Sexual risk**						
No risk	2734	95.1(93,96.6)	3227	97.5(94.7,98.9)	5961	96.4(94.3,97.7)
Risk	153	4.9(3.4,7)	66	2.5(1.1,5.3)	219	3.6(2.3,5.7)
**Physically active**						
No	2430	82.6(77.4,86.8)	2794	86.9(82.2,90.5)	5224	84.8(80.5,88.2)
Yes	554	17.4(13.2,22.6)	536	13.1(9.5,17.8)	1090	15.2(11.8,19.5)
**Bullying**						
No	1334	44.3(39.9,48.9)	1838	53.8(50,57.6)	3172	49.3(45.6,53)
Yes	1505	55.7(51.1,60.2)	1427	46.2(42.4,50)	2932	50.7(47,54.4)
