# Supplementary material for: Correlates of bullying victimization among school adolescents in Nepal: Findings from 2015 Global School-Based Student Health Survey Nepal
Source: PLoS One. 2020 Aug 19;15(8):e0237406. doi: 10.1371/journal.pone.0237406 (PMC7444580; doi:10.1371/journal.pone.0237406)
Supplement: S3 Table — (PDF) [file pone.0237406.s003.pdf]

Table S3. Prevalence of school bully-victims among school going adolescents of Nepal.

Variables	Total Number	Weighted Count	Prevalence	95%CI
**Age**				
Early adolescent	3130	1514	50.43	45.08,55.77
Late adolescent	3028	1453	51.45	47.54,55.35
**Sex***				
Male	2839	1505	55.67	51.09,60.15
Female	3265	1427	46.17	42.4,49.98
**Felt lonely***				
Ever	2102	1245	61.52	56.74,66.08
Never	3971	1671	45.38	41.4,49.43
**Anxiety***				
Ever	2209	1349	64.97	60.44,69.25
Never	3955	1618	43.65	39.39,48.0
**Considered suicide**				
Yes	752	455	60.84	54.95,66.44
No	5329	2460	49.02	44.71,53.35
**Attempted suicide***				
Yes	544	391	72.9	67.24,77.9
No	5629	2587	48.47	44.59,52.36

**Involved in physical fight*** **				
Yes	2339	1578	70.14	65.78,74.17
No	3845	1399	38.34	34.7,42.11
**Missed school feeling unsafe*** **				
Yes	2345	1380	61.66	57.15,65.98
No	3784	1562	43.25	38.63,47.99
**Missed school without permission*** **				
Yes	1644	971	60.81	56.38,65.07
No	4406	1942	46.91	42.73,51.13
**Smoking *** **				
Yes	328	240	75.22	67.81,81.39
No	5740	2671	49.2	45.43,52.98
**Smokeless tobacco use *** **				
Yes	290	217	76.05	66.72,83.42
No	5884	2756	49.41	45.79,53.04
**Alcohol use *** **				
Yes	265	193	72.51	60.68,81.84
No	5814	2712	49.42	45.67,53.18
**Overweight**				
Yes	315	162	53.15	43.39,62.67
No	5886	2826	50.72	47.22,54.22

**Underweight**				
Yes	598	312	53.6	48.58,58.55
No	4910	2324	50.08	46.56,53.59
**Sexual risk behavior ****				
Yes	213	129	65.14	54.95,74.12
No	5769	2737	50.26	46.44,54.07
**Physically active**				
Yes	1068	501	49.36	42.29,56.45
No	5035	2440	51.19	47.36,55.01
**Total**	**6201**	**2988**	**50.86**	**47.23,54.47**

*p < 0.05, **p < 0.01, ***p < 0.001
