# Supplementary material for: Correlates of bullying victimization among school adolescents in Nepal: Findings from 2015 Global School-Based Student Health Survey Nepal
Source: PLoS One. 2020 Aug 19;15(8):e0237406. doi: 10.1371/journal.pone.0237406 (PMC7444580; doi:10.1371/journal.pone.0237406)
Supplement: S4 Table — (PDF) [file pone.0237406.s004.pdf]

Table S4. Multivariable analysis of health risk behaviors of school bully-victims among school going adolescents of Nepal.

Variables	Crude OR	95% CI	Adjusted OR*	95% CI
**Age (Ref:Early adolescent)**				
**Late adolescent**	1.04	0.81,1.33	0.97	0.74,1.28
**Sex (Ref:Male)**				
**Female**	0.68	0.58,0.80	0.79	0.67,0.93
**Smoking (Ref:No)**				
Yes	3.13	2.13,4.61	1.43	0.91,2.26
**Smokeless tobacco use (Ref:No)**				
Yes	3.25	2.07,5.11	2.05	1.15,3.65
**Alcohol use (Ref:No)**				
Yes	2.70	1.55,4.68	1.1	0.74,1.62
**Overweight (Ref:No)**				
Yes	1.10	0.79,1.55	0.99	0.71,1.39
**Underweight (Ref:No)**				
Yes	1.15	0.91,1.46	1.13	0.83,1.54
**Sexual Risk Behavior (Ref:No)**				
Yes	1.85	1.20,2.85	0.87	0.51,1.47
**Involved in physical fight (Ref:No)**				
Yes	3.78	3.06,4.66	3.64	2.94,4.51

Physically active (Ref:No)				
Yes	0.93	0.70,1.24	0.96	0.69,1.34

*Adjusted for age, sex, smoking, smokeless tobacco use, alcohol use, overweight, underweight, sexual risk behavior, involved in physical fight and physically active
