# Supplementary material for: Correlates of bullying victimization among school adolescents in Nepal: Findings from 2015 Global School-Based Student Health Survey Nepal
Source: PLoS One. 2020 Aug 19;15(8):e0237406. doi: 10.1371/journal.pone.0237406 (PMC7444580; doi:10.1371/journal.pone.0237406)
Supplement: S5 Table — (PDF) [file pone.0237406.s005.pdf]

Table S5. Multivariable analysis of mental health behaviors of school bully -victims among school going adolescents of Nepal.

Variables	Crude OR	95% CI	Adjusted OR*	95% CI
**Age (Ref:Early adolescent)**				
**Late adolescent**	1.04	0.81,1.33	0.88	0.67,1.15
**Sex (Ref:Male)**				
**Female**	0.68	0.58,0.80	0.63	0.53,0.75
**Loneliness (Ref:Never)**				
Ever	1.92	1.58,2.34	1.36	1.12,1.64
**Anxiety(Ref:Never)**				
Ever	2.39	1.93,2.96	2.04	1.65,2.52
**Considered suicide(Ref:No)**				
Yes	1.62	1.16,2.26	0.94	0.7,1.24
**Attempted suicide(Ref:No)**				
Yes	2.86	2.12,3.86	2.08	1.54,2.81
**Missed school feeling unsafe(Ref:No)**				
Yes	2.11	1.67,2.67	1.72	1.34,2.21
**Missed school without permission(Ref:No)**				

Yes	1.76	1.43,2.16	1.48	1.17,1.87

*Adjusted for loneliness, anxiety, considered suicide, attempted suicide, missed school feeling unsafe and missed school without permission
