# Supplementary material for: Correlates of bullying victimization among school adolescents in Nepal: Findings from 2015 Global School-Based Student Health Survey Nepal
Source: PLoS One. 2020 Aug 19;15(8):e0237406. doi: 10.1371/journal.pone.0237406 (PMC7444580; doi:10.1371/journal.pone.0237406)
Supplement: Attachment — Submitted filename: Response to Reviewers.docx [file pone.0237406.s006.docx]

**Reviewer #1:**

**Methods:**

**Reviewer’s comment**: The authors have mentioned that they have used a nationally representative data. However, no information is provided on the regions from which the schools were selected. Were the selected schools government or private schools, were these schools selected across the country or confined to a certain area alone?
What were the characteristics of the schools? For a country like Nepal, schools' characteristics vary widely between regions as well as between sectors. The problems faced by students from one of the most expensive school in Kathmandu isn't comparable to the students who need to walk for hours to reach the school in some remote parts of the country. Thus, more information on the schools are necessary.
The authors did acknowledge in the limitations section that certain information about schools are missing. In that case, the authors should provide an elaborate explanation on how it is still a nationally representative data despite the lack of information in the methods section. Or provide more details about the schools.
The method section has elaborated about the school survey as a primary data collection study. However, as this is a study based on secondary data analysis, it would be important for the authors to also elaborate the methods on how the data was retrieved? Were all the data from the survey analysed? Was the sample size calculated for this secondary study? Were there any inclusion or exclusion criteria for selecting the data for this secondary analysis study? In short, please focus on the methods used for this part of the study rather than on the already published survey.

**Author’s response**: This study is secondary data analysis of Global School based Health Survey Nepal and data set of this survey can be accessed from WHO website (<https://www.who.int/ncds/surveillance/gshs/nepaldataset/en/>).  This study includes all the data of the data set, so, no any separate sample size was calculated especially for this study. In the first stage of sampling, all the schools containing any of 7-11 class across the country were listed and 74 schools were selected with probability proportional to school enrolment size. According to the researchers involved in the study, schools from both government and private schools were included in the survey but GSHS report hasn’t mentioned about it. Similarly, while drawing schools, ecological belt (hill, mountain and terai) was also considered. From each ecological belt, 25 schools were taken. However, the dataset doesn’t contain information on it.

**Results:**

**Reviewer’s comment**: It would have been better if Table 1. was about the general characteristics instead of the variables. The table on variables could have been added as a supplementary file.
In Table 1. I see all the variables are dichotomized but for sensitive issues such as mental health (anxiety and loneliness), e.g. being anxious all the times, sometimes or most of the time have different implications. It loses information on the severity if always, sometimes and most of the time are grouped into one category. So either it should be grouped as ''ever'' and ''never''. Or it would have been better if the authors created dummy variables for each severity such as for anxiety- sometimes=1, rest =0, then always=1, rest=0, and so on to fit in each severity in the analysis. If it’s not possible, please acknowledge it as a limitation that dichotomizing the variables could have lead to potential loss of information and it doesn't reflect the severity.

**Author’s response:** Revised as suggested (included characteristics of participants, coded mental factors as ever and never)

**Reviewer’s comment**: In the multivariable analyses, why weren't the demographic characteristics such as age and sex adjusted? They are important confounders and it is important to control them in multivariable analysis given that the sex had already shown some significant association with bullying.
Ideally, if possible the schools or clusters of schools too should have been controlled in the analysis. As there are many schools, it is understandable that all schools cannot be controlled. Instead they could be grouped into similar schools such as government vs private schools or schools by region and controlled accordingly because schools are important confounders affecting the results for the reasons mentioned above. If it is impossible for this study to control school please provide an explanation.

**Author’s response:** Regarding controlling school related features (public vs private, ecological belts) in multivariable analysis; the data set do not provide the information about the type of schools and ecological belts in which schools were located. So, this make impossible to adjust school related features. We adjusted age and sex in multivariable analysis.

**Other Minor comments**
**Reviewer’s comment**: Line 38: Please spell out DALYS when it appears for the first time in the manuscript

**Author’s response:** Revised as suggested

**Reviewer’s comment**: Line 40: Please spell out SDI

**Author’s response:** Revised as suggested

**Reviewer #2:**

**Reviewer’s comment**: The overall manuscript seems technically sound. The keywords are missing in the abstract which should have been mentioned. The manuscript has been prepared in an intelligible fashion and standard English has been used. But framing of sentences can be made more concise in overall manuscript so that the article is more interesting for readers. The statistical analysis has been performed appropriately and vigorously. Operational definitions for overweight and underweight not mentioned under variable description. Recommendations can be made more specific towards associated factors and variables.

**Author’s response:** Revised as suggested

Operational definitions for overweight: >+1SD from median for BMI by age and sex

Operational definitions for underweight: <-2SD from median for BMI by age and sex
