# Supplementary material for: Correlates of bullying victimization among school adolescents in Nepal: Findings from 2015 Global School-Based Student Health Survey Nepal
Source: PLoS One. 2020 Aug 19;15(8):e0237406. doi: 10.1371/journal.pone.0237406 (PMC7444580; doi:10.1371/journal.pone.0237406)
Supplement: Attachment — Submitted filename: Response to Reviewers.docx [file pone.0237406.s007.docx]

***Reviewer’s comment***: Have the authors made all data underlying the findings in their manuscript fully available?

***Author’s response***: The dataset supporting the conclusions of this article is open access data and is available in the WHO website: https://www.who.int/ncds/surveillance/gshs/nepaldataset/en/

***Reviewer’s comment***: it would be better if the authors could also include some of the responses to the comments as part of the manuscript in the **limitations** section. Such as the authors mentioned that "According to the researchers involved in the study, schools from both government and private schools were included in the survey but GSHS report hasn’t mentioned about it." This statements could be included (not entirely but briefly and rephrased) in the limitation section.
***Author’s response***: Included as suggested in limitation section (This survey lacks the data based on geographical and socio-economic information, **schools’ characteristics (government or private schools)** and other factors that might determine bully.)

***Reviewer’s comment***: The authors have discussed the key findings elaborately with reference to global literature on bullying. However, it would be insightful if the authors could also elaborate a little on potential solutions **to address bullying in schools for a country like Nepal**. The authors concluded that "the findings of this study provide valuable insights for planning an effective approach in preventing bullying at schools". It would be insightful to understand what these "effective approaches" could be. A paragraph focusing on recommendations could help this paper provide more depth given that the paper uses a nationally representative data.
***Author’s response***: Revised as suggested in conclusion section

***Reviewer’s comment***:

The manuscript needs proofreading.
Lines 39-42- The sentence is too long and difficult to follow. It can be broken down into two different sentences. Also "in" is missing before 2017 (line 41).
There are prepositions missing in some other parts of the manuscript as well.

***Author’s response***:

Revised as suggested. Proofreading is also done on the manuscript.
